# A case of *Trueperella pyogenes* causing prosthetic joint infection

**DOI:** 10.5194/jbji-6-47-2020

**Published:** 2020-12-01

**Authors:** Tariq Azamgarhi, Simon Warren

**Affiliations:** 1 Pharmacy Department, Royal National Orthopaedic Hospital NHS Trust, Brockley Hill, Stanmore, HA7 4LP, UK; 2 Department of Infectious Diseases, Royal Free Hospital, Pond Street, Hampstead, NW3 2QG, UK

## Abstract

We present the first reported case of prosthetic joint infection caused by *Trueperella pyogenes*. This animal pathogen rarely causes human infection. Our patient was successfully treated with single-stage exchange and 12 weeks of rifampicin and moxifloxacin.

## 1 Introduction

*Trueperella pyogenes* is a well-known opportunistic pathogen of livestock and other animals. Only a few sporadic cases of human infection have been reported, and these are usually associated with occupational exposure to host animals such as cattle and pigs in farmers and abattoir workers.

## 2 Case report

A 78-year old Chinese female was referred to the Royal National Orthopaedic Hospital (RNOH) with a 6-month history of worsening buttock pain following a fall onto her right hip. The pain was intermittent and unresponsive to analgesia or physiotherapy, and she was unable to walk 10 m without a walking aid. Eighteen months prior she underwent an uncomplicated right Corail Pinnacle total hip replacement (THR) for osteoarthritis resulting in a well-healed scar and good function.

Her past medical history included hypertension; L2/L3 and L5/S1 nerve root decompression; hysterectomy 3 months after the initial THR; and two tympanoplasties of the left ear but was otherwise healthy. She did not smoke or drink alcohol and she reported a penicillin allergy with a rash.

**Figure 1:**
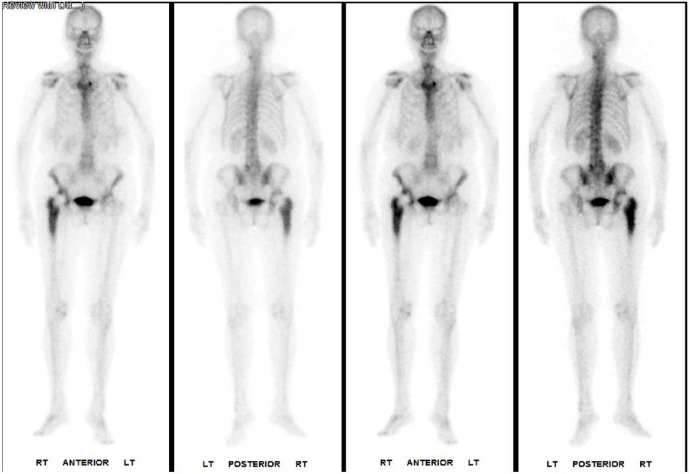
Single-photon emission computed tomography (SPECT) showing increased signal around the right hip and signs of chronic periosteal reaction around the femur.

Examination revealed a well-healed posterolateral scar with no erythema, swelling or discharge but was otherwise unremarkable. The patient denied having fevers, shivering or acute infections since hip replacement surgery. Blood tests showed a raised C-reactive protein (CRP) of 21 mg/L (normal range 0–5 mg/L) and erythrocyte sedimentation rate (ESR) of 50 mm/h (normal range 0–12 mm/h) and a neutrophil count of $4.5\times 10^{9}$ cells/L (normal range 1.5–8.$0\times 10^{9}$ cells/L). A plain film of the pelvis demonstrated chronic periosteal reaction in the right proximal femur suspicious for infection. A single-photon emission computed tomography (SPECT) scan confirmed the plain film findings and showed an increased signal around the right hip (Fig. 1). Following discussion at the bone infection multidisciplinary team (MDT) meeting, aspiration was recommended.

Fluoroscopic aspiration of the right hip yielded viscous fluid and tissue samples that were sent for microbiological analysis. *Staphylococcus warnerii *was isolated in one sample. In the other *Trueperella pyogenes* grew after 48 h of culture and was identified using matrix-assisted laser desorption ionization-time of flight (MALDI-TOF) mass spectrometry (Bruker Microflex™ with Biotyper 3.1™). Leukocyte counts were unable to be performed on the fluid sample.

Based on the rarity of the organism cultured and uncertainty around the diagnosis of prosthetic joint infection (PJI), the MDT recommended repeat aspiration for synovial leukocyte counts and culture. This demonstrated a leukocyte count of 14 440 cells/cu.mm (>4200 cells/µL) with 70 % polymorphonucleocytes (>65 % neutrophils). *Trueperella pyogenes* was again cultured, confirming the diagnosis of PJI according to published criteria (Osmon et al., 2013; Parvizi et al., 2018; Signore et al., 2019). The isolate was sent to the reference laboratory at Public Health England, Colindale, UK, which confirmed the identification and sensitivity to amoxicillin, erythromycin, fusidic acid, teicoplanin, vancomycin, gentamicin, moxifloxacin, rifampicin, linezolid, co-trimoxazole and resistance to tetracycline.

Subsequently the MDT recommended surgical treatment with a single-stage revision of the hip which was performed 26 months after the index operation. Operative findings included acetabular and femoral components that were loose and easily explanted with minimal bone loss. Five deep intraoperative samples were sent. A definitive Corail Pinnacle revision THR was re-implanted with vancomycin-impregnated bone graft (Osteomycin). Empiric antibiotic therapy with intravenous teicoplanin (10 mg/kg 12-hourly for three doses then 24-hourly) and two doses of amikacin (15 mg/kg once daily) was commenced. All five samples grew *Trueperella pyogenes*, and one grew *Staphylococcus warnerii*, which was considered a contaminant.

By day 11 after surgery the wound was dry, she was mobilising well, and a plain film demonstrated a well-positioned prosthesis. She was switched to an oral regimen of rifampicin 450 mg twice daily and moxifloxacin 400 mg daily and discharged on day 13.

She tolerated antimicrobial therapy well with no adverse effects and bloods, including full blood count, Urea and electrolytes and liver function tests remained normal throughout. At clinical review after 12 weeks there was complete healing of the wound with excellent function and antibiotics were stopped. Twenty-four months after surgery she continues pain-free with no concern of relapse.

## 3 Discussion

*Trueperella pyogenes* is a non-motile, facultatively anaerobic, pleomorphic Gram-positive bacillus. It grows well on blood agar, typically exhibiting a zone of beta-haemolysis after 24 h of growth. Identification was historically based on morphology, a negative catalase assay and biochemical profile using commercially available systems (e.g. API Coryne); however, more recent techniques include MALDI-ToF, which give rapid and reliable results when compared with molecular methods (Hajizin et al., 2012; Randall et al., 2015). In our case, *Trueperella pyogenes* was identified using MALDI-TOF.

**Figure 2:**
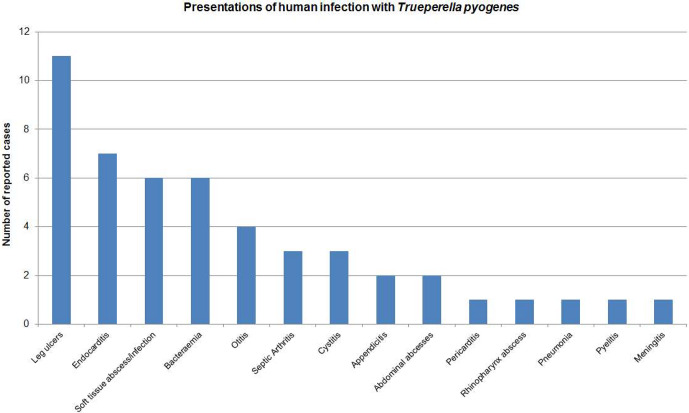
Results of a PubMed search using the terms “human”, “infection”, “*Trueperella pyogenes*”, “*Aranobacterium pyogenes*” and “*Actinomyces pyogenes*”. Forty-nine cases of human infection were identified.

Originally described and named *Corynebacterium pyogenes* in 1903, it has been reassigned to different genera several times since: to Actinomyces in 1982 based on morphological and chemotaxonomic criteria; then Arcanobacterium in 1997 based on 16S rRNA gene sequence analysis; and finally, with several other species, into the newly described genus Trueperella in 2011 after further phylogenetic analysis (Rzewuska et al., 2019; Yassin et al., 2011).

*Trueperella pyogenes* possesses several potential virulence factors, but their significance in pathogenesis is unclear. The ability to form biofilm has been demonstrated by in vitro studies and may be important in PJI (Zhao et al., 2013). *Trueperella pyogenes* colonises mucous membranes of wildlife and livestock (Rzewuska et al., 2019). It can be found in the udder, urogenital and upper respiratory tracts of cattle, and pigs. It is an opportunistic pathogen that, especially in combination with Gram-negative anaerobic bacteria, is known to cause suppurative or necrotising infection including cutaneous eruptions, mastitis, endometritis, liver abscesses, pneumonia, and septic arthritis. Infections in cattle are important economically due to loss of milk yield, reduced reproductive efficiency, and the occasional need to cull infected animals (Rzewuska et al., 2019).

In contrast, it has not been isolated as part of the normal human flora. A PubMed search using “human infection”, “*Trueperella pyogenes*” and its previous names as search terms identified 49 reports since 1946 involving various body sites (Fig. 2), but this is the first report of PJI. Reported infection is typically acute or life-threatening, usually occurring in individuals with underlying chronic illness such as liver failure, diabetes and lung cancer. Infection may be linked to contact with livestock or their environment and is often reported as a presumed zoonosis (Plamondon et al., 2007). Our patient's presentation was less typical as she had chronic localised infection and no underlying chronic illness.

The aetiology in our patient is uncertain. She had no history of acute infection to suggest haematogenous spread. She denied consuming unpasteurised dairy products, so digestive transmission is unlikely. In cattle summer mastitis has been associated with transmission by a biting fly, *Hydrotaea irritans* (Rzewuska et al., 2019), and in humans leg ulceration has been associated with transmission by Oriental-eye flies in Thai children (Kotrajaras and Tagami, 1987). Both seem unlikely in our case. Although human colonisation has not been demonstrated, it has been hypothesised in infection in patients with no obvious occupational exposure (Plamondon et al., 2007; Kotrajaras and Tagami, 1987). Our patient was born in Hong Kong and worked as a farmer tending cattle from an age of 8 to 20. Subsequently she moved to China where she lived in close contact with pig livestock, before emigrating to the UK in 1965 and working in kitchens until retirement 10 years before presentation. A thorough history elicited no further animal contact or recent travel. The prior history of occupational exposure with long-term colonisation and subsequent opportunistic infection 50 years later is a possibility. In the absence of a preceding acute illness, contamination at implantation with late presentation 12 months later seems the likeliest aetiology.

The indolent presentation made it difficult to establish a definite diagnosis of PJI. Variation between published PJI definitions, particularly use of joint aspirates, synovial leukocyte counts and biomarkers, made pre-operative diagnosis uncertain. Repeat aspiration was useful in our case, where we had a high index of suspicion for PJI but growth of an unusual organism in a single pre-operative sample, as further culture of the same organism met criteria for three international definitions of PJI.

No specific evidence exists to guide the optimal surgical strategy for treatment of *Trueperella pyogenes* PJI. Consistent with published guidance, the MDT considered it reasonable to recommend a single-stage strategy based on knowledge of the infecting organism and sensitivity to rifampicin and fluoroquinolones in a patient with no significant immunocompromise or poor soft tissues (Osmon et al., 2013).

There are few data to guide the choice of antimicrobial treatment of Trueperella infection. The available data from animal studies demonstrate conflicting in vitro activity against penicillin, gentamicin, and tetracyclines. The overuse of these antimicrobials in agriculture may account for this variation (Rezanejad et al., 2019; Ribeiro et al., 2015). Rifampicin and moxifloxacin were used based on susceptibility testing, high oral bioavailability, good bone penetration, and biofilm activity (Osmon et al., 2013). Following a single-stage exchange, 12-week duration of antibiotic treatment is generally recommended (Osmon et al., 2013; Parvizi et al., 2018). Local vancomycin may have contributed to the successful outcome.

In conclusion, we report the first case of PJI caused by *Trueperella pyogenes*. In the absence of recent animal contact, colonisation after prior occupational exposure with subsequent infection is the most likely aetiology. This case highlights the importance of multidisciplinary input in the diagnosis and treatment of PJI, especially where there is an unusual organism and an indolent presentation.

### Ethical statement

Consent was received from the patient prior to submission for publication.

## Data Availability

No data sets were used in this article.
